# Serotype-specific Cardiac Involvement in Pneumococcal Pneumonia

**DOI:** 10.1093/cid/ciaa1434

**Published:** 2020-09-22

**Authors:** Keith P Klugman, Charles Feldman

**Affiliations:** 1 Department of Global Health, School of Public Health, Emory University, Atlanta, Georgia, USA; 2 Department of Medical Microbiology, School of Pathology, University of the Witwatersrand Medical School, Johannesburg, South Africa; 3 Pneumonia Program, Bill and Melinda Gates Foundation, Seattle, Washington, USA; 4 Department of Internal Medicine, Faculty of Health Sciences, University of the Witwatersrand, Johannesburg, South Africa

**Keywords:** pneumococcus, pneumonia, serotype, cardiac disease

**(See the Major Article by Africano et al on Volume 72, Issue 11, 1 June 2021, pages e711–9.)**

The involvement of the heart as part of invasive pneumococcal disease (IPD) has not been nearly as widely recognized as the more feared complication of pneumococcal meningitis. A number of lines of evidence, however, suggest that not only is cardiac disease an important risk factor for IPD, being present in 37% of adults with IPD older than 65 years of age in the United States [1], but the converse may also be true, and cardiac involvement is increasingly being described in current case series of adult pneumonia as an important component of IPD disease severity, and may also play a role in the longer term consequences of IPD.

Bacterial pneumonia, including pneumococcal pneumonia, has been recognized as a significant risk factor for acute coronary syndrome, with an extraordinary risk ratio of 47.6 demonstrated in the 15 days postpneumonia in a relatively recent self-controlled case series [2]. The pathogenesis of these cardiac events post–pneumococcal pneumonia is not clearly understood, and may reflect instability of coronary thrombus following the inflammatory events of a pneumonia episode, but have recently been suggested to reflect microinvasion of the heart itself by the pneumococcus, which has been demonstrated in mice, monkeys, and in some humans [3]. The experimental occurrence of these lesions in animal models is restricted to certain highly invasive serotypes, including serotypes 2, 3, and 4 [4]. Not only is a community-acquired pneumonia episode a risk factor for cardiovascular disease in the acute phase postinfection, but heart failure may be increased for as long as 10 years after the pneumonia episode [5].

It is within this context that Africano and coworkers [6] have published in this issue of *Clinical Infectious Diseases* an important further contribution to our knowledge of cardiac complications of pneumonia and IPD by recording cardiac events, defined in their study as major adverse cardiovascular events (MACE), comprising either new or worsening heart failure, arrhythmias, and/or a myocardial infarction in 23% of a series of 310 patients with IPD, including 28% of those admitted for community-acquired pneumonia. The occurrence of MACE in this study was associated with a significantly increased incidence of mechanical ventilation and in-hospital mortality [6].

A perusal of the remarkable book by Heffron [7] that details in over 1400 references the literature published during the pre–antibiotic era on pneumococcal pneumonia recognizes the severity of bacteremia (p 690) and arrhythmias (p 722) as poor prognostic signs in severe cases of lobar pneumonia [7]. Heffron’s conclusion from the pre–antibiotic era is, however, that while myocarditis may be “frequently present in patients dying of lobar pneumonia, myocardial changes during the illness are generally insignificant, and, as already mentioned (p 492–494 and p 544–546), pneumonia does not cause heart disease (p 608)” [7]. There is, further, no significant discussion of long-term consequences of pneumonia, with the suggestion that, while recurrence may be more common, later episodes may be less severe (p 539) [7].

It appears, therefore, that the long-term cardiovascular consequences of pneumococcal pneumonia may be more apparent in current case series, where most patients are elderly, with significant underlying cardiovascular morbidity, and all are treated with antibiotics, than among younger adults described during the pre–antibiotic era.

Indeed, in the study that found cardiac microlesions in animals and humans with IPD [3], while antibiotic treatment resulted in healing of cardiac microlesions over 1 week, there was cardiac scarring, which may account for long-term cardiac complications [3]. Antibiotic treatment itself may play a role in the release of pneumolysin, the pore-forming activity of which, in the mouse model, plays an essential role in the pathogenesis of these microlesions [3, 8]. In an in vitro model, exposing cardiomyocytes to pneumolysin-expressing pneumococci caused dose-dependent cardiomyocyte dysfunction and death, exacerbated further by antibiotic treatment, due to increased pneumolysin release [8]. These authors describe a model whereby high pneumolysin concentrations, with large pore formation, lead to extensive cardiomyocyte lysis, but sublytic, low-dose, small pore-forming pneumolysin concentrations cause calcium influx into the cardiomyocytes with substantial calcium overload, resulting in mechanical and electrical disturbances that could explain contractile dysfunction and rhythm disturbances [8].

A remarkable aspect of the study in this issue by Africano and coworkers [6] is that, in contrast to usual practice where serotyping of isolated pneumococci is rarely performed, the 310 patients included in the study were those from whom serotyping data were available and showed in a multivariable analysis the association of serotypes 3 and 9N with new cardiovascular abnormalities during the illness (*P* = .04).

Given the number of serotypes detected, and the small numbers due to individual serotypes, as mentioned by the authors, this observation should be considered as hypothesis generating, rather than a conclusive study. Nonetheless, despite the inclusion of serotype 3 in the 13-valent conjugate vaccine, this serotype has not declined in incidence globally, and there is no evidence to date that serotype 9V, included in all licensed pneumococcal conjugate vaccine (PCV) products, cross-protects against serotype 9N.

It is thus quite reasonable to make the argument that an improved serotype 3 vaccine remains a future priority and that serotype 9N may have an additional claim to inclusion in future PCV formulations, given the role that these invasive serotypes may play in the cardiovascular consequences of pneumococcal pneumonia. It has further been recently shown that the lung invasiveness of a serotype 1 strain of pneumococcus is associated with its ability to release pneumolysin during autolysis [9].

These data also raise the issue of a re-consideration of the inclusion of pneumolysin as a carrier in future PCVs.

## Note

***Potential conflicts of interest.*** C. F. reports grants, personal fees, and from Pfizer and personal fees and from MSD (Merck), outside the submitted work; he has been assisting the authors of the *Clinical Infectious Diseases* manuscript, upon which the comment/editorial is based, with another, separate study on the pneumococcus. K. P. K. reports no potential conflicts.
